# Investigating Nuisance Effects Induced in EEG During tACS Application

**DOI:** 10.3389/fnhum.2021.637080

**Published:** 2021-05-28

**Authors:** Romain Holzmann, Judith Koppehele-Gossel, Ursula Voss, Ansgar Klimke

**Affiliations:** ^1^GSI Helmholtzzentrum für Schwerionenforschung GmbH, Darmstadt, Germany; ^2^Vitos Hochtaunuskliniken, Friederichsdorf, Germany; ^3^Department of Psychology, J. W. Goethe-Universität, Frankfurt am Main, Germany; ^4^Department of Psychiatry, Heinrich-Heine-Universität Düsseldorf, Düsseldorf, Germany

**Keywords:** EEG, tACS, modulation, hardware demonstrator, artifact removal

## Abstract

Transcranial alternating-current stimulation (tACS) in the frequency range of 1–100 Hz has come to be used routinely in electroencephalogram (EEG) studies of brain function through entrainment of neuronal oscillations. It turned out, however, to be highly non-trivial to remove the strong stimulation signal, including its harmonic and non-harmonic distortions, as well as various induced higher-order artifacts from the EEG data recorded during the stimulation. In this paper, we discuss some of the problems encountered and present methodological approaches aimed at overcoming them. To illustrate the mechanisms of artifact induction and the proposed removal strategies, we use data obtained with the help of a schematic demonstrator setup as well as human-subject data.

## 1. Introduction

Low-current electrical stimulation of the human brain is a powerful technique developed in applied and experimental neuroscience (Herrmann et al., 2013; Paulus et al., 2013). Especially transcranial alternating current stimulation (tACS) is a unique form of non-invasive brain stimulation in which sinusoidal currents are delivered to the scalp to affect mostly cortical neurons. Among other things, tACS has been used for the entrainment of brain activity at specific frequencies, aiming at a synchronization of cortical oscillators (Helfrich et al., 2014b; Witkowski et al., 2016) and at localized increases in specific targeted frequencies, e.g., alpha (Zaehle et al., 2010) or gamma power (Voss et al., 2014). Moreover, Elyamany et al. (2020) showed in a recent review that tACS may also have the potential to reset disturbed brain oscillations, and thereby be able to support pharmacotherapy and psychotherapy in various mental disturbances, like obsessive-compulsive disorder (OCD), depression, bipolar disorder, dementia, and attention-deficit/hyperactivity disorder (ADHD). In many research studies, the neural response is inferred from the characteristics of the recorded electroencephalogram (EEG). Also from a clinical viewpoint, it would be of special interest to determine whether a regional modulation of neuronal circuits takes place during the tACS stimulation itself. This might be useful, both as a predictor and a quantitative correlator of the clinical efficacy of the treatment. However, analyzing and interpreting the EEG during concurrent tACS are very demanding tasks because of the extensive artifacts induced in the data, in both the time and frequency domains. To remove the stimulation artifacts from the recorded EEG or, at least, minimize their impact in the subsequent analysis, various schemes have been proposed; see Caldwell et al. (2020) for a brief review. Common approaches are to either (1) filter the artifact in the frequency domain (Helfrich et al., 2014a; Kohli and Casson, 2019), (2) subtract an artifact template in the time domain (Helfrich et al., 2014b; Voss et al., 2014; Caldwell et al., 2020), or (3) apply spatial filters constructed by modal decomposition of the EEG signals (Neuling et al., 2017; Guarnieri et al., 2020; Haslacher et al., 2020; Vosskuhl et al., 2020). Spatial filtering relies on concurrent information from a sufficiently large number of EEG sites and it requires, therefore, a high-density EEG montage. Note that combinations of several methods have been used as well; for example, Fehér et al. (2017) have subtracted a moving-average template followed by a principal component analysis (PCA). While most of these artifact-removal techniques are now quite well-established, recent investigations by Noury et al. (2016) and Noury and Siegel (2017) have revealed that unavoidable quasi-periodic physiological processes, like heartbeat and respiration, can induce additional, non-linear effects in the EEG through a rhythmic modulation of the main tACS frequency. Indeed, Noury et al. observed in EEG data both an amplitude modulation (AM) and a phase modulation (PM) of the stimulation artifact, which they attributed to periodic changes of the body-tissue impedance. They also posited that these changes would be caused by the pulsating blood flow and the regular breathing movements imprinting a modulation at frequencies in the range of 1–2 and 0.2–0.5 Hz, respectively, and thus provoking a corresponding spread in the EEG frequency spectrum. As a consequence, the possible occurrence of such spreads must be carefully considered in any artifact-removal procedure used, especially when the stimulation is applied in the frequency band targeted by the investigation.

In the present paper, we focus on the AM of the stimulation artifact in EEG recordings. We first recapitulate how the modulation of the signal amplitude produces side bands in the EEG power spectrum and we also illustrate the effect with a schematic simulation. We then suggest a novel cleaning procedure that allows to remove the modulation from the recorded EEG signals. We provide proof of principle by applying the proposed scheme to mock EEG data measured with the help of a demonstrator, i.e., an experimental setup based on a simple phantom scalp. Finally, we demonstrate the procedure and its performance on data recorded from a human subject in a realistic laboratory setting. In doing so, we focus on stimulation frequencies applied in the EEG low and high gamma bands (30–140 Hz) where modulation artifacts are most liable to impact the overall descending intrinsic EEG power spectrum.

## 2. Amplitude Modulation and Demodulation

### 2.1. AM Signal Modulation

AM of a sinusoidal signal introduces periodic changes of its maximum value as a function of time, i.e., it imprints those changes onto the signal envelope. In AM broadcasting, the information transported by the radio signal, e.g., voice, music or data, is hence encoded in its envelope. To illustrate the modulation process in tACS, let us first consider a sinusoidal signal *V*(*t*), of amplitude *A*_∘_, frequency *f*_*s*_, and phase *φ*_*s*_ = 0, expressed as a function of time *t* by

(1) $$\begin{array}{l} V_{\circ }\left(t\right)=A_{\circ }\sin\omega_{s}t, \end{array}$$

where *ω*_*s*_ = 2*πf*_*s*_. Introducing furthermore a periodic change of the amplitude at a frequency *f*_*m*_, realized here as *A*(*t*) = *A*_∘_[1 + *m* cos *ω*_*m*_*t*], we arrive at the following, amplitude-modulated signal

(2) $$\begin{array}{l} V\left(t\right)=A\left(t\right)\sin\omega_{s}t=A_{\circ }\left[1+m\cos\omega_{m}t\right]\sin\omega_{s}t, \end{array}$$

with *ω*_*m*_ = 2*πf*_*m*_ and *m* ∈ [0, 1] being the so-called modulation index. Applying the trigonometric identity 2 sin* A* cos *B* = sin(*A* + *B*) + sin(*A* − *B*), this expression can be rewritten as

(3) $$\begin{array}{l} V\left(t\right)=A_{\circ }\sin\omega_{s}t+\frac{1}{2}mA_{\circ }\left[\sin\left(\omega_{s}+\omega_{m}\right)t+\sin\left(\omega_{s}-\omega_{m}\right)t\right]. \end{array}$$

Note that, due to the modulation, two additional terms appear at frequencies *f*_*s*_ ± *f*_*m*_, i.e., equally spaced above and below the main signal frequency. For a more complex periodic modulation signal, e.g., specified as $A\left(t\right)=\overset{\infty }{\underset{k=1}{\sum }}a_{k}\cos k\omega_{m}t$, the relation expressed by Equation (2) can be generalized^1^ to the form

(4) $$\begin{array}{l} V\left(t\right)=A\left(t\right)\sin\omega_{s}t=A_{\circ }\left[1+m\overset{\infty }{\underset{k=1}{\sum }}a_{k}\cos k\omega_{m}t\right]\sin\omega_{s}t, \end{array}$$

which in turn expands into the expression

(5) $$\begin{array}{l} V(t)=A_{\circ }\sin\omega_{s}t+\frac{1}{2}mA_{\circ }[\sum_{k=1}^{\infty }a_{k}\sin(\omega_{s}+k\omega_{m})t\\ \;+\sum_{k=1}^{\infty }a_{k}\sin(\omega_{s}-k\omega_{m})t]. \end{array}$$

It appears from Equation (5) that the full Fourier spectrum characterizing the modulation signal *A*(*t*) is added on either side of the central frequency. The same result follows directly from the convolution theorem of the Fourier transformation F. In fact, by applying the operator F to Equation (4), one finds

(6) $$\begin{array}{l} ℱ[V(t)]=ℱ[A(t)\times \sin\omega_{s}t)]=ℱ[\sin\omega_{s}t]⊗ℱ[A(t)]\\ \;=\delta (f-f_{s})⊗ℱ[A(t)], \end{array}$$

where ⊗ stands for the convolution, or folding, operation. The right side of Equation (6) corresponds to the comb-spectrum expressed by Equation (5).

### 2.2. AM Signal Demodulation

Conversely, by demodulating an amplitude-modulated signal, one can recover the information encoded in its envelope; mathematically, this corresponds to reversing the modulation operation expressed by Equation (2). In more practical terms, e.g., in an AM radio receiver, the envelope can be retrieved by multiplying the (amplified) captured signal *V*(*t*) with a local oscillator signal, tuned and phase-locked to the central frequency *f*_*s*_ of *V*(*t*), giving the product

(7) $$\begin{array}{l} V(t)\times \sin\omega_{s}t=A(t)\sin\omega_{s}t\times \sin\omega_{s}t\\ \;=A(t)\sin^{2}\omega_{s}t\\ \;=\frac{1}{2}A(t)[1-\cos2\omega_{s}t]. \end{array}$$

After having removed the 2 *ω*_*s*_ harmonic with an appropriate low-pass filter, this procedure yields the low-frequency AM signal *A*(*t*). This demodulation principle, called product detector, is typically implemented in analog radio receivers. A more effective separation of the harmonics is achieved with higher-order product detectors, using the following scheme:

(8) $$\begin{array}{l} V(t)\times \sin\omega_{s}t\cos^{2}\omega_{s}t=A(t)\sin^{2}\omega_{s}t\cos^{2}\omega_{s}t\\ \;=\frac{1}{8}A(t)[1-\cos4\omega_{s}t]. \end{array}$$

Again, the signal to be demodulated is multiplied with a phase-locked local oscillator signal as well as with the square of a 90° phase-shifted derivation of the latter, leading now to a high-frequency term at 4 *ω*_*s*_ that can be filtered more easily from the low-frequency envelope *A*(*t*). Note that yet more sophisticated schemes can be devised, but are then implemented most conveniently by software in a digital receiver.

In the present context, namely the modeling of artifacts generated by tACS in electrophysiological signals, the data are usually available as digitized signal samples that can readily be subjected to more sophisticated signal analysis procedures. In particular, the analytical representation of a signal can be computed as $V_{a}\left(t\right)=V\left(t\right)+iH\left[V\left(t\right)\right]$, where H represents the Hilbert transformation operator (Bendat and Piersol, 2010). From this, the envelope *A*(*t*) can be directly obtained as the norm of *V*_*a*_(*t*), namely

(9) $$\begin{array}{l} A\left(t\right)=|V_{a}\left(t\right)|=\sqrt{V{\left(t\right)}^{2}+H{\left[V\left(t\right)\right]}^{2}}. \end{array}$$

**Figure 1:**
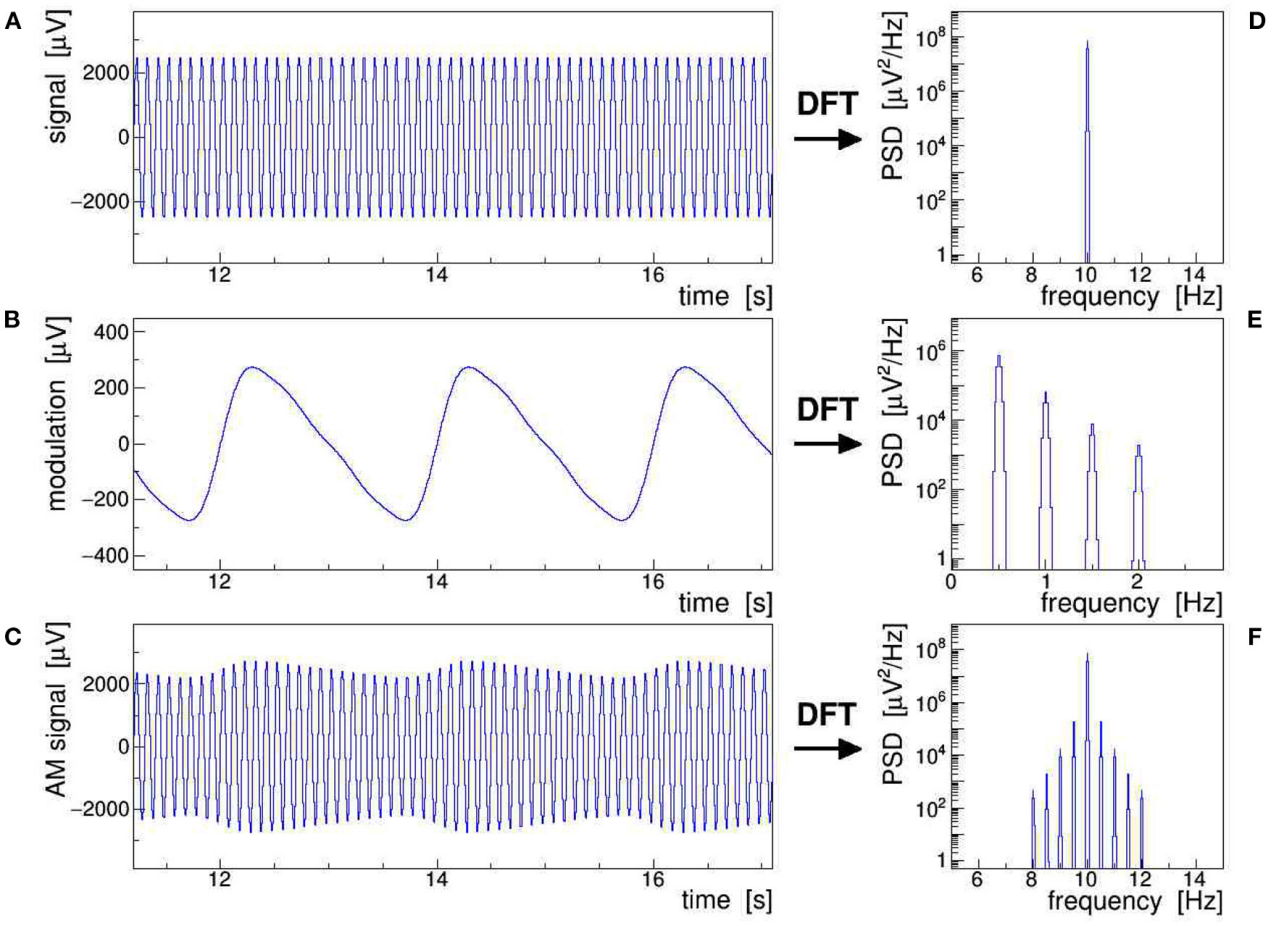
Schematic representation of an amplitude-modulated sinusoidal signal: a 10-Hz sine wave **(A)** is modulated with a 0.5 Hz saw tooth **(B)** resulting in the amplitude modulation (AM) signal **(C)**. The power spectral density (PSD) of the corresponding discrete Fourier transforms are shown in frames **(D)**–**(F)**. This figure also exemplifies Equation (6), which states that the multiplication of **(A)** with **(B)**, giving **(C)** in the time domain, corresponds to a convolution of **(D)** with **(E)**, giving **(F)** in the frequency domain.

Figure 1 illustrates all of the AM concepts discussed above by showing a stable 10-Hz sine wave **(A)** modulated with a slow saw tooth **(B)**. The latter was approximated as the sum of a 0.5-Hz sine and contributions of its first four harmonics at 1, 1.5, 2, and 2.5 Hz. The modulation index was set to *m* = 0.1, i.e., large enough to make the AM envelope clearly visible in the modulated signal **(C)**. The power spectral density (PSD) distributions of the respective discrete Fourier transform (DFT), presented in frames **(D)**–**(F)**, show that **(F)** results indeed from folding **(D)** with **(E)**, as expressed by Equation (6). The original saw tooth can be retrieved from the modulated signal by applying either of Equations (7)–(9), combined with proper low-pass filtering. Here, we have posited that the modulation of the signal is stationary, i.e., that its ensemble-averaged moments (mean, variance, etc.) remain constant over the Fourier-transformed signal segment, while ergodicity is not explicitly required (Bendat and Piersol, 2010). This assumption is justified when analyzing epochs of EEG signals shorter than the typical time scales of slow impedance changes caused by sweating or drying conductive paste, as well as of sporadic shifts due to, e.g., posture changes.

**Figure 2:**
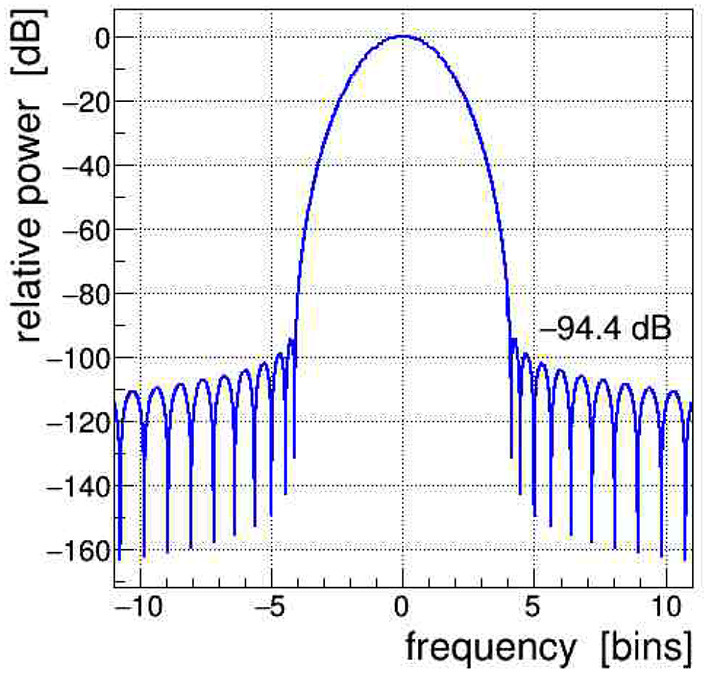
Frequency spectrum of the Kaiser–Bessel windowing function, with parameter *α* = 4, applied in the discrete Fourier transform (DFT); frequency is expressed in bins of width 1/*T*, with *T* being the time span of the Fourier-transformed signal. The power leakage of this window is very low, all side lobes are at < −94 dB, i.e., a factor of nearly 10 orders of magnitude below the central lobe.

All signals shown in Figure 1 were Fourier transformed into the frequency domain using a Kaiser–Bessel windowing function with parameter *α* = 4; its frequency response is shown in Figure 2 and its detailed characteristics are given in Heinzel et al. (2002). It is indeed very important to control the side-lobe power leakage and keep it well below the expected level of the side-band artifacts induced in the EEG data. We selected this particular window type because it offers a large side-lobe suppression of −94.4 dB together with a reasonably good frequency resolution corresponding to a width of the main lobe at its base of Δ*f* = ±4.1 frequency bins.^2^

### 2.3. Simulation of EEG Signals With Concurrent tACS

**Figure 3:**
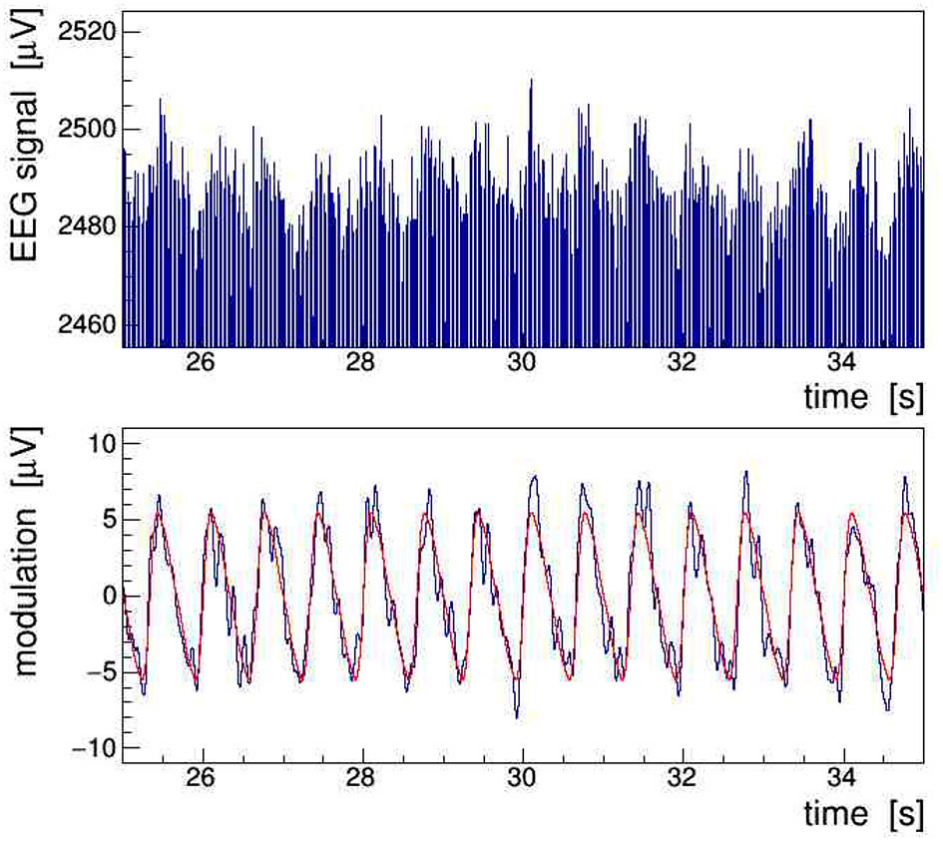
Simulation of an amplitude-modulated 40-Hz sinusoidal signal of 5 mV_pp_ added on top of a random baseline of pink noise. (Top) Upper edge of the full signal visualizing the 1.5 Hz saw-tooth modulation at 10 *μ*V_pp_ depth. (Bottom) Envelope of the signal recovered by demodulation (black) shown with the overlaid true modulation signal (red).

**Figure 4:**
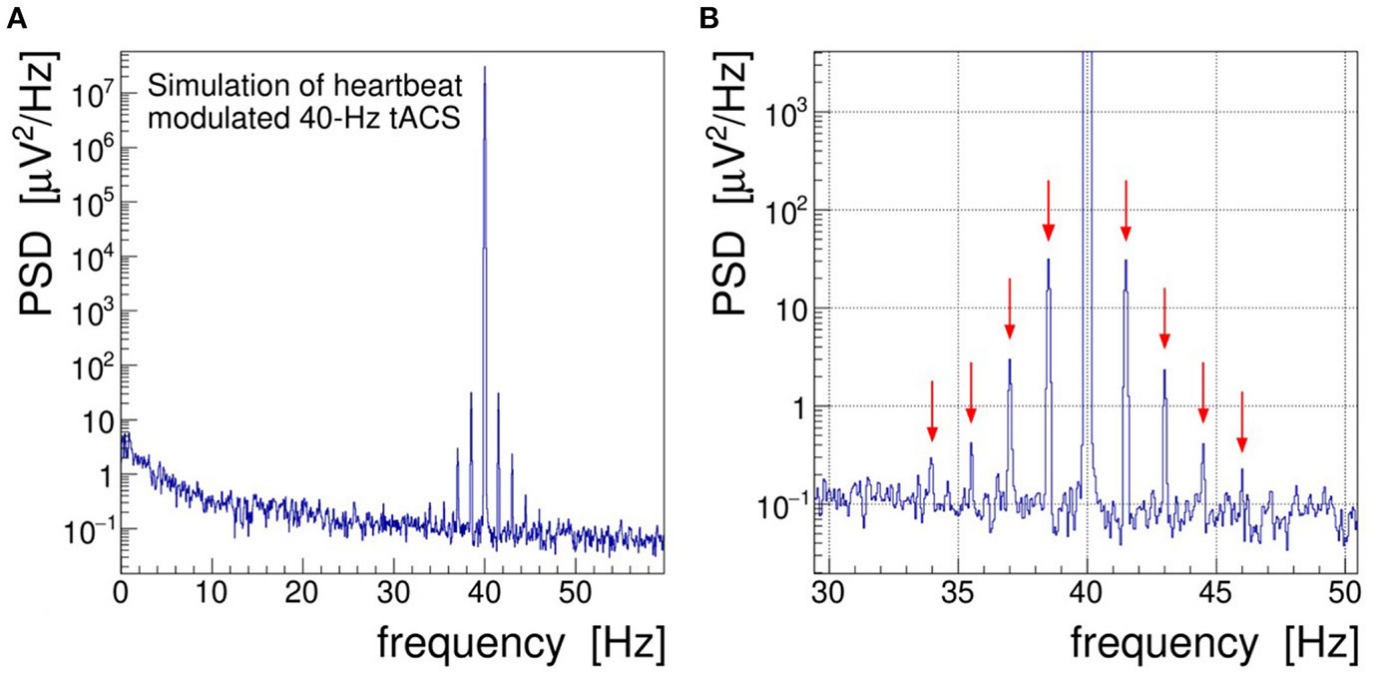
**(A)** Power spectral density (PSD) distribution obtained by Fourier transforming the simulated signal shown in Figure 3. In this simulation, the electroencephalogram (EEG) signal is realized by sampling a pink noise distribution and the transcranial alternating current stimulation (tACS) signal is represented by a 40-Hz sine wave modulated at the level of *m* = 0.2% with a 1.5 Hz saw tooth. **(B)** Zoom into the gamma frequency band; red arrows mark the side peaks arising at *f* = 40 ± n × 1.5 Hz through the amplitude modulation of the tACS voltage.

In order to achieve a more realistic demonstration of the artifacts induced in EEG by concurrent tACS, we have run a computer simulation in which the intrinsic brainwave signal was mimicked by sampling pink noise, i.e., a random distribution with its power falling off at −10 dB/frequency decade, and the tACS signal was added as a 40-Hz sinusoidal signal amplitude-modulated with a 1.5 Hz saw-tooth beat.^3^ In addition, a closer to reality (see section 4), that is, a much weaker modulation index of 0.002 was chosen. As shown in Figure 3, the characteristic fluctuations of the signal AM envelope remain visible, but only barely so, because of the random nature of the underlying pink noise. In the frequency domain, however, the modulation artifacts appear very prominently. The PSD spectrum resulting from a DFT of the simulated signal, presented in Figure 4, shows that the modulation-induced side-band power is substantial when compared with the underlying brainwave power. Consequently, in a quantitative analysis of tACS-induced changes of the EEG, not only the stimulation artifact itself will have to be removed with very high precision, but also its side bands will eventually have to be cleaned. Notice that in this simulation, the leakage of the Kaiser–Bessel window function is negligible as it stays more than two orders of magnitude below the pink noise spectrum.

## 3. Hardware Demonstrator of Stimulation Artifacts

### 3.1. Setup and Data Recording

When moving from simulations to real EEG data, in a first step, we aimed at investigating the stimulation artifacts in a realistic, yet fully controlled laboratory setting. To do so, we have assembled a hardware demonstrator with the following components:

- a scalp phantom, realized as coarse-grained finite-element 4.7 kΩ resistor network;
- a variable impedance—to mimic periodic effects, e.g., heartbeat and respiration—implemented as a light-dependent resistor (LDR) rhythmically illuminated by a light-emitting diode (LED);
- a digital signal generator, model FY6800 (FeelTech, China)—to drive the LED;
- a tACS device, the DC Stimulator Plus (neuroConn GmbH, Germany)—to inject a sinusoidal current into the phantom;
- a 64 EEG channel + 8 AUX channel 24-bit recorder, actiCHamp (Brain Products GmbH, Germany)—to acquire multichannel data;
- a 4-channel digital storage oscilloscope, DS1104Z (Rigol Technologies, China)—used for testing purposes only, but not on a human subject.

**Figure 5:**
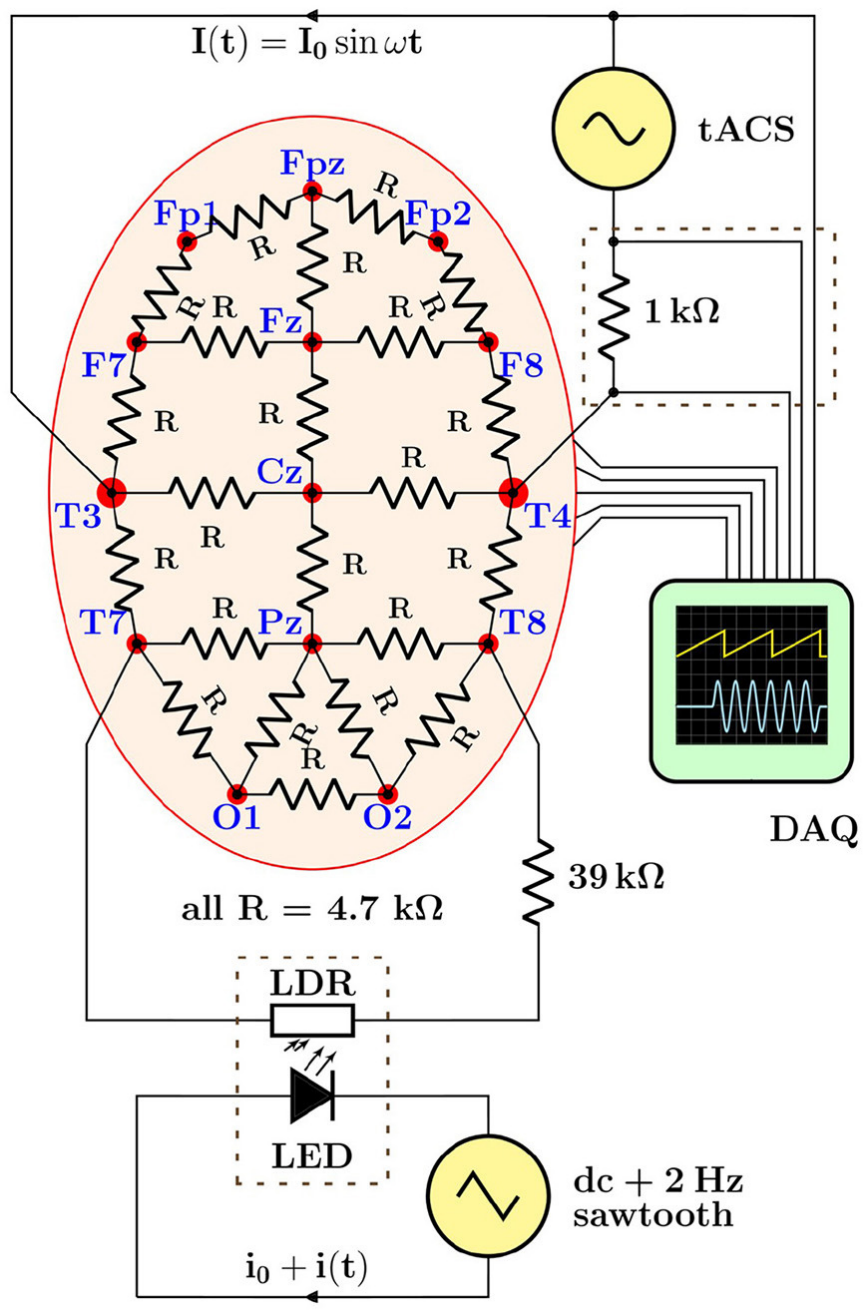
Schematic overview of the hardware demonstrator designed to generate electroencephalogram (EEG) data with typical transcranial alternating current stimulation (tACS) artifacts in a realistic but fully controllable laboratory setting. The lower dashed rectangle stands for the light-tight enclosure holding the light-emitting diode (LED)/light-dependent resistor (LDR) setup used to induce rhythmic impedance changes; the upper dashed rectangle represents the breakout box from which tACS voltage and current signals are derived, see text for details.

In this setup, shown schematically in Figure 5, the data acquisition (DAQ) system used to digitize EEG signals was either a state-of-the-art 72-channel EEG recorder or, for some basic tests, a digital storage oscilloscope. The main rationale behind realizing a hardware demonstrator was that it allowed to acquire data with the full instrumental chain—stimulator, electrode leads, and EEG recorder—in the actual laboratory environment, i.e., including the real power-line interferences (50 Hz and harmonics), amplifier noise and non-linearities, as well as stimulator noise and harmonics. We refrained from using a 3d multi-layer head phantom, like the ones discussed by Owda and Casson (2020) and Vosskuhl et al. (2020), as our aim was to obtain sample EEG data for our testing purposes only. Furthermore, we were also not concerned with volume vs. scalp conduction or capacitive electrode impedance effects. Our resistive-net phantom provided the means to record in a controlled and reproducible, yet sufficiently realistic way the data required to design and validate adequate artifact removal procedures.

**Figure 6:**
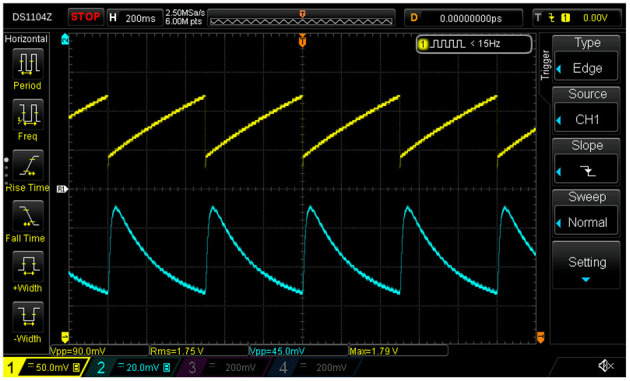
Scope traces of the saw tooth (here 2-Hz, ΔU = 0.1 V) light-emitting diode (LED) driving voltage (yellow) and the beat-induced impedance changes in the scalp phantom of the demonstrator (blue).

Demonstrator data were recorded with a basic montage consisting of four EEG leads connected to the phantom (Fp1, Fp2, Fpz as ground, and Cz as reference) as well as derivations of the tACS voltage and current signals (see section 4 and upper dashed box in Figure 5) fed into two of the bipolar AUX channels of the recorder; all signals were sampled at a rate of 1 kHz. The stimulator was set to deliver a 40-Hz sinusoidal stabilized current of 0.5 mA_pp_ through a total impedance of 4.2 kΩ across sites T3 and T4. The modulation of the scalp impedance was achieved by connecting the LDR/LED pair across sites T7 and T8, and driving the LED with the programmable signal generator that provided 0.2 V saw-tooth pulses at 2 Hz repetition rate on top of a 2 V DC pedestal; the modulation intensity could be easily set by adjusting the amplitude of the saw tooth. The driving voltage of the LED and the resulting impedance changes, visualized as voltage drop across the LDR, are visible in the scope traces shown in Figure 6. Notice, in particular, that the characteristic response of the LED/LDR couple leads to slightly non-linear variations of the mock scalp impedance.

**Figure 7:**
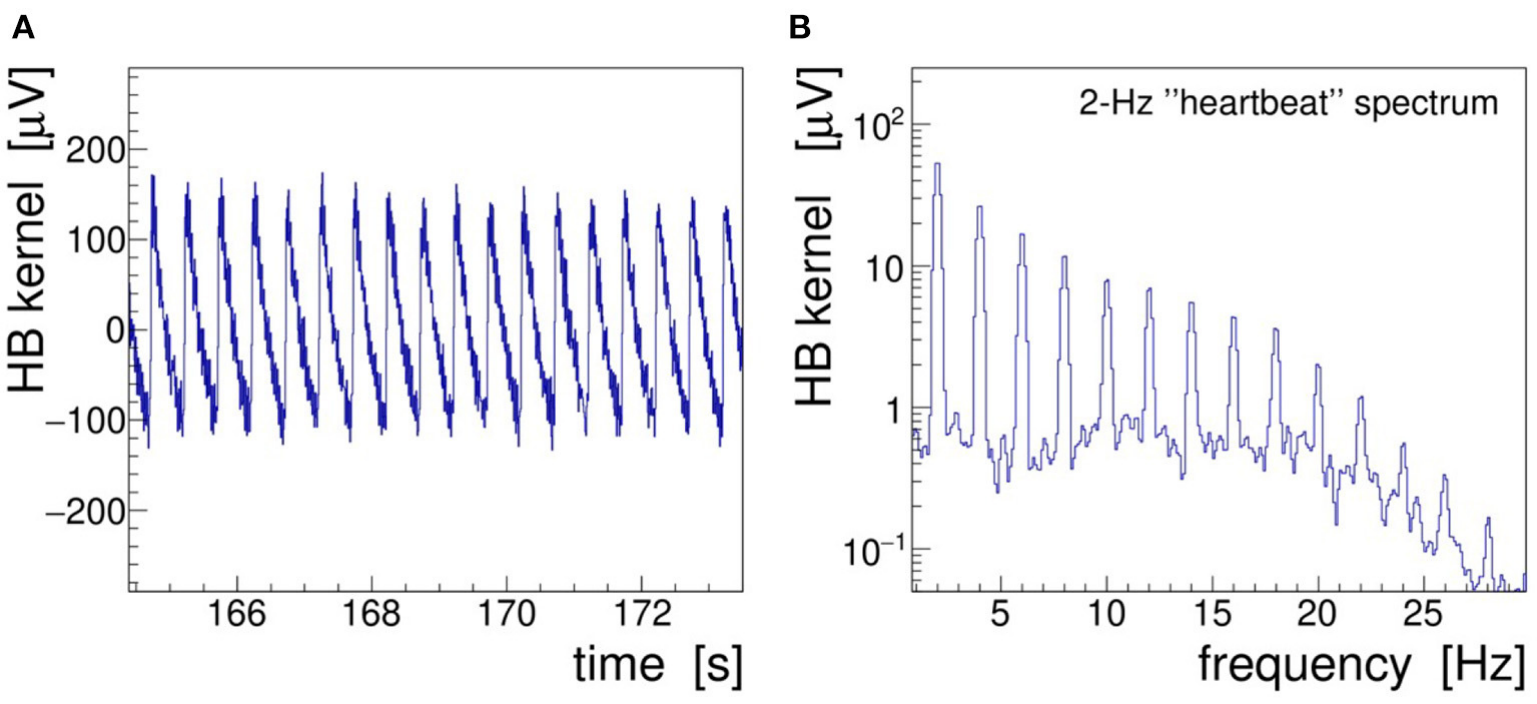
Heartbeat modulation kernel retrieved by demodulation of the demonstrator electroencephalogram (EEG) signal Fp1 recorded during 40-Hz transcranial alternating-current stimulation (tACS) application and 2-Hz saw-tooth impedance modulation. **(A)** Demodulated signal in the time domain and **(B)** r.m.s. spectrum of the modulation kernel in the frequency domain.

In the following text and pictures, we designate the induced 2-Hz modulation as “heartbeat” or, in brief, “HB.” A 10-s long segment of demonstrator data is presented in Figure 7 showing the demodulated, filtered, and detrended signal envelope—called modulation kernel by Noury et al. (2016)—both in the time **(A)** and frequency **(B)** domains. The frequency spectrum was obtained by low-pass filtering (25 Hz, 32nd-order, zero-delay Butterworth) and Fourier-transforming (*α* = 4 Kaiser–Bessel window) the kernel. As discussed in section 2.2, this filter is needed to suppress the harmonics of the stimulation signal; it also removes operational noise picked up in the laboratory environment (mostly 50 Hz power line interferences).

### 3.2. Artifact-Removal Procedures

Here, we propose a two-step procedure to remove the tACS artifacts from the recorded EEG data. In a first step, we remove the AM-induced side-band power by reversing^4^ the amplitude-modulation process described in section 2.1. In a second step, we delete the main and by far dominant stimulation artifact by subtracting a properly scaled and phase-shifted segment of the concurrently recorded tACS current signal. These cleaning steps are applied one by one to each EEG channel of interest and to each data episode of interest, meaning that all EEG channels are processed individually and independently. The advantage of such a procedure is that it can be applied universally—in particular, to few-channel montages—with the sole condition that the tACS current signal is also available in the recorded data sets.

**Figure 8:**
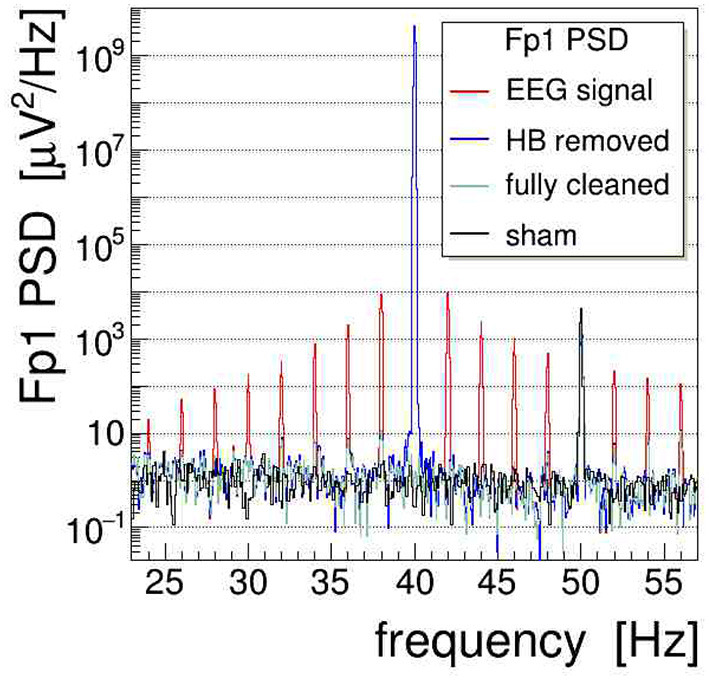
Power spectral density (shown in red, 0.05 Hz bins) of the demonstrator electroencephalogram (EEG) signal Fp1 during 40-Hz transcranial alternating current stimulation (tACS) application and amplitude modulation with a 2-Hz saw-tooth “heartbeat” (HB). The modulation causes substantial and far-reaching spread of power to both sides of the stimulation frequency. By applying the two-step cleaning procedure outlined in the text, first, the amplitude modulation (AM)-induced artifact is strongly suppressed (blue spectrum) and, second, the main artifact at 40 Hz is removed, resulting in a fully cleaned power spectral density (PSD) spectrum (overlaid in green). For comparison, the cleaned PSD obtained without stimulation, i.e., under sham condition, is also shown (black). See text for a discussion of the persisting power in the 50-Hz peak.

In order to achieve our first goal, i.e., the removal of AM-induced side-band power, we rewrite the amplitude *A*(*t*) of the tACS-induced potential in a given EEG signal as

$$\begin{array}{l} A\left(t\right)=A_{\circ }+K\left(t\right)=A_{\circ }\left[1+m\hat{K}\left(t\right)\right], \end{array}$$

where *A*_∘_ is the constant amplitude of the unmodulated signal and $K\left(t\right)=mA_{\circ }\hat{K}\left(t\right)$ represents the modulation of the amplitude. In this expression, the normalized kernel $\hat{K}\left(t\right)$ encodes the time dependence of the signal envelope and, as already introduced in Equation (2), *m* expresses the relative depth of the modulation. From the measured EEG signal, *K*(*t*) is obtained by (1) AM demodulation (see section 2.2), (2) detrending of the retrieved envelope to remove the constant term, and (3) low-pass filtering to suppress the harmonics of the stimulation signal. With *K*(*t*) available individually for each EEG channel and episode of interest, we can apply a multiplicative correction to the measured signal. The basic idea is to divide out the kernel under the assumption that the modulation is a small perturbation only, i.e., *m* ≪ 1; this is indeed justified as data reveal typically values of *m* ≈ 10^−4^ − 10^−2^ (see Noury et al., 2016 and section 4). To expand on this, let us write the total EEG signal *V*_sig_ observed at a given scalp site as a superposition of intrinsic and induced signals

$$\begin{array}{l} V_{\text{sig}}\left(t\right)=V_{\text{eeg}}\left(t\right)+V_{\text{tacs}}\left(t\right)=V_{\text{eeg}}\left(t\right)+A\left(t\right){Î}_{\text{tacs}}\left(t\right), \end{array}$$

where *V*_eeg_(*t*) is the intrinsic EEG signal of interest (including environmental and instrumental noise) and *V*_tacs_(*t*) is the modulated voltage induced at this site by the applied stimulation current *I*_tacs_(*t*); here, the artifactual signal is expressed as a function of the normalized stimulation current *Î*_tacs_(*t*). Both signals, *V*_sig_(*t*) and *I*_tacs_(*t*), are measured and an estimate of *A*(*t*), and hence $m\hat{K}\left(t\right)$, is obtained by demodulation of *V*_sig_(*t*). We proceed to remove the modulation artifact from the recorded EEG signal by applying sample by sample the following operation:

(10) $$\begin{array}{l} V_{\text{sig}}(t)\Rightarrow V_{\text{sig}}^{\text{(1)}}(t)=\frac{V_{\text{sig}}(t)}{\left[1+(m+\Delta m)\hat{K}(t+\Delta t)\right]}\\ \;=\frac{V_{\text{eeg}}(t)+A(t){Î}_{\text{tacs}}(t)}{\left[1+(m+\Delta m)\hat{K}(t+\Delta t)\right]}\\ \;≃V_{\text{eeg}}(t)+\frac{A(t){Î}_{\text{tacs}}(t)}{\left[1+(m+\Delta m)\hat{K}(t+\Delta t)\right]}\\ \;≃V_{\text{eeg}}(t)+A_{\circ }{Î}_{\text{tacs}}(t), \end{array}$$

where $V_{\text{sig}}^{\text{(1)}}\left(t\right)$ stands now for the AM-corrected signal after cleaning step one. The approximation is valid in most practical cases, as the division only marginally affects the intrinsic EEG signal itself when *m* ≪ 1. The parameters Δ*t* and Δ*m* represent small adjustments of *t* and *m*, respectively (Δ*t*/*t*, Δ*m*/*m* ≪ 1). They are needed to achieve an optimal subtraction of the artifact: the time adjustment Δ*t* corrects for possible small phase differences between *V*_sig_(*t*) and *V*_tacs_(*t*), caused by the hardware or the analysis (e.g., different filters applied), whereas adjustments of the modulation index Δ*m* correct for slow drifts of the modulation depth during averaging over a number of time spans. Best values of both Δ*t* and Δ*m* are determined in a regression procedure^5^ set up, such as to optimally remove the AM-induced side-band power in the data segment of interest. This requires computing the Fourier transform repeatedly within the regression loop, as the optimization is controlled by the ratio of integrated power in the side-bands to the power in the main peak. To minimize this ratio, we have used a robust simplex algorithm (Nelder and Mead, 1965), which does not require the gradients of the functional to be minimized. The efficacy of this procedure is demonstrated on our Fourier-transformed (20-s data segments, *α* = 4 Kaiser–Bessel window) phantom data in Figure 8

where the PSD distributions are compared before and after applying the first cleaning step. One clearly sees that the side-band artifacts are reduced by up to four orders of magnitude (i.e., ≤ 40 dB) in the vicinity of the main peak. The efficacy decreases slowly when moving further away from the stimulation frequency, say by ±20 Hz, although in these regions the side-peak power is fading out anyway. This particular behavior is not surprising, however, as the regressed parameters in Equation (10) are, by construction of the minimized functional, most sensitive to the lower harmonics of the AM kernel. It remains to be explored whether a more sophisticated filtering of the kernel can help to further improve the cleaning. In Figure 8, we also show the PSD obtained under a “sham” condition, i.e., for data recorded while the stimulator was switched off. Comparing sham with the clean-signal PSD, we conclude that the cleaning procedure introduces no bias in the power spectrum. A further point to notice is that the PSD at 50-Hz remains largely unaltered due to power-line noise interfering at this particular frequency; full removal can only be achieved by applying a dedicated notch filter.

The second step, i.e., the removal of the main tACS artifact, is achieved on the AM-cleaned signal $V_{\text{sig}}^{\text{(1)}}\left(t\right)$ by subtracting sample by sample a properly scaled and phase-shifted copy of the normalized stimulation signal *Î*_tacs_(*t*), resulting in the fully cleaned signal $V_{\text{sig}}^{\left(2\right)}\left(t\right)$, as follows:

(11) $$\begin{array}{l} V_{\text{sig}}^{\left(1\right)}\left(t\right)\Rightarrow V_{\text{sig}}^{\left(2\right)}\left(t\right)=V_{\text{sig}}^{\left(1\right)}\left(t\right)-n_{\circ }I_{\text{tacs}}\left(t+\Delta t\right). \end{array}$$

The optimal scaling factor *n*_∘_ and time shift Δ*t* are again determined with the help of a simplex regression by minimizing the residual stimulation power summed over the data segment of interest. Figure 8 shows that the second cleaning step completely removes the huge stimulation artifact from the PSD distribution. This is quite remarkable considering that to cleanly subtract an artifact more than nine orders of magnitude (i.e., ≥90 dB) bigger than the intrinsic EEG baseline requires very high precision on the parameters of Equation (11). In fact, a more detailed investigation revealed that the length of the signal segment on which these parameters are optimized impacts the efficacy of the second cleaning step: using a too large segment results in not subtracting the artifact completely, while using a very short one entails local over-subtraction, possibly producing a dip at 40 Hz. A certain degree of oversubtraction is to be expected as, in the present case, the method targets one specific frequency, just like a digital notch filter would do.

The full, two-step cleaning procedure is eventually applied to the recorded EEG data in a signal-by-signal and epoch-by-epoch manner. As the regression coefficients are recomputed for each epoch, slow trends in impedance change, caused by drying electrode paste, sweating, etc., will not impair the correction. Furthermore, the described procedure has the advantage of also being applicable to few-electrode EEG montages.

## 4. Application to Human Subject Data

We now turn to our investigation of AM effects on multichannel EEG data recorded from a healthy subject during application of tACS. We first describe the experiment, then we assess the size of stimulation-induced artifacts in the EEG power spectra, and finally we evaluate the efficacy of the cleaning procedures introduced in the previous section.

### 4.1. Experimental Techniques

A multichannel EEG was recorded from a healthy subject (age range 20–25) using an electrode cap instrumented with 61 active scalp electrodes according to the 10–10 positioning system; the ground electrode was placed on the forehead at position Fpz and all signals were referenced to electrode Cz. An electrooculogram (EOG) was taken with passive electrodes from the outer canthi of both eyes and supraorbitally to the left eye; likewise, passive electromyogram (EMG) electrodes were fixed at the chin. Both, EOG and EMG leads were connected to bipolar AUX inputs of the EEG recording device (actiCHamp, Brain Products, Germany). Electrocardiogram (ECG) electrodes were fixed below the left clavicle and left costal arch of the subject, and a pressure sensor belt was put on, delivering ECG and respiration signals, respectively, to further bipolar AUX inputs. Data of all EEG and AUX channels were filtered (0.16 Hz high-pass and 1,000 Hz low-pass at 12-dB/octave) and digitized at a sampling rate of 10 kHz.

Stimulation current was applied to the subject through four 3.5 × 4 cm^2^ conducting silicone-rubber electrodes placed across frontal and temporal sites F5/F7 and TP9, respectively, F6/F8 and TP10, and connected pair-wise to the output jacks of a tACS stimulator (DC Stimulator Plus, neuroConn, Germany). The current leads were thereby routed through a custom-built breakout box, allowing to derive voltage and current signals that were both fed into additional AUX inputs of the EEG recorder. The current derivation was taken as the voltage drop over a 1 kΩ resistor (see upper dashed box in Figure 5) while still warranting full galvanic isolation of the subject during the experiment.

The experiment consisted of recording a number of few-minute episodes of EEG and AUX data while applying a sinusoidal tACS current of 0.6 mA peak-to-peak (electrode impedance of 6.8–7.6 kΩ) at frequencies of either 40, 70, or 100 Hz for 3 min at a time. Recording started and stopped about 20–30 s before and after stimulation, respectively. Care was taken to avoid driving the EEG amplifiers into saturation, which would otherwise have resulted in distorted or even clipped signals. From the hardware side, currents up to about 1.5 mA would have been tolerable but, as they provoked excessive skin irritations on the subject, we refrained from using them. The implied current limitation is also typical for sleep studies employing concurrent tACS–EEG where one wants to avoid induced awakenings (Voss et al., 2014). For all recordings, the subject was seated in an upright position, immobile, awake, and with eyes closed.

For the offline analysis, the data were low-pass filtered at 333 Hz, notch-filtered at the power-line frequency and at its odd harmonics (*f*= 50, 150, 250, and 350 Hz, Δ*f*= 0.5 Hz), and then decimated by a factor 10 to a sampling rate of 1 kHz. The EOG, EMG, and ECG signals were furthermore low-pass filtered at 25 Hz to block the stimulation frequency; filtering was not required, however, for the RESP signal derived from the respiration belt piezo sensor which had no electrical contact with the subject's skin.

To localize individual heartbeat and breathing events in time, we have applied a QRS-complex detector (Pan and Tompkins, 1985; Kohler et al., 2002) to the ECG signal and a feature search to the RESP signal. The event times were furthermore synced with the nearest zero-crossing of the recorded tACS signal, shifting each event by an appropriate amount of samples.^6^ This syncing is necessary to preserve the phase relation of the stimulation current when averaging EEG segments over a sequence of heartbeats or breathing events.

### 4.2. Assessing the Stimulation Artifacts

The characteristic frequency dependence of a subject's natural EEG spectrum is thought to follow roughly the one of pink noise, with the consequence that any modulation artifacts are more easily visible for stimulation frequencies in the gamma band or above where the intrinsic EEG power is lowest. Therefore, we focus our discussion on the EEG data recorded with 40, 70, or 100 Hz tACS.

**Figure 9:**
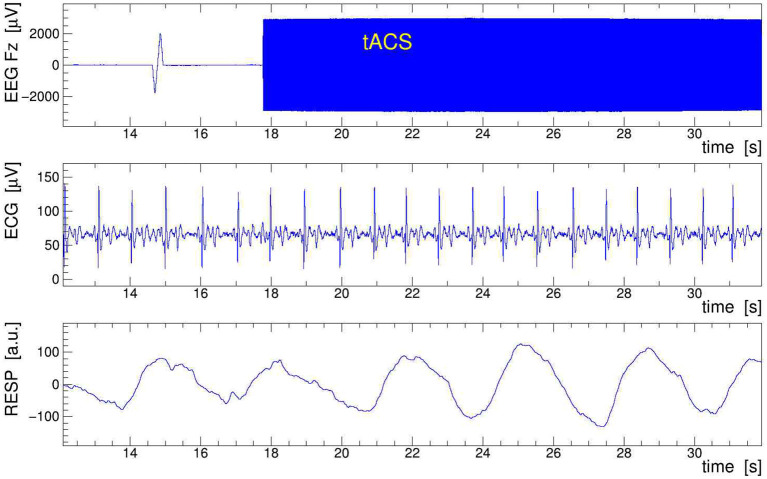
Sample of electroencephalogram (EEG) data taken on human subject while 100-Hz transcranial alternating current stimulation (tACS) was applied. Shown are 20 s of the EEG signal Fz (top frame), the electrocardiogram (ECG) signal (middle frame), and the respiration belt signal (bottom frame). In Fz, note the switch-on spike at *t* = 15 s and the onset of stimulation at *t* = 18 s, resulting in a larger than 100-fold increase of the signal amplitude. The low-pass filtered ECG and RESP signals show the subject's heartbeat and respiration events, respectively; they serve to extract the event-averaged amplitude modulation (AM) kernels that are used to remove the modulation from the EEG signal.

**Figure 10:**
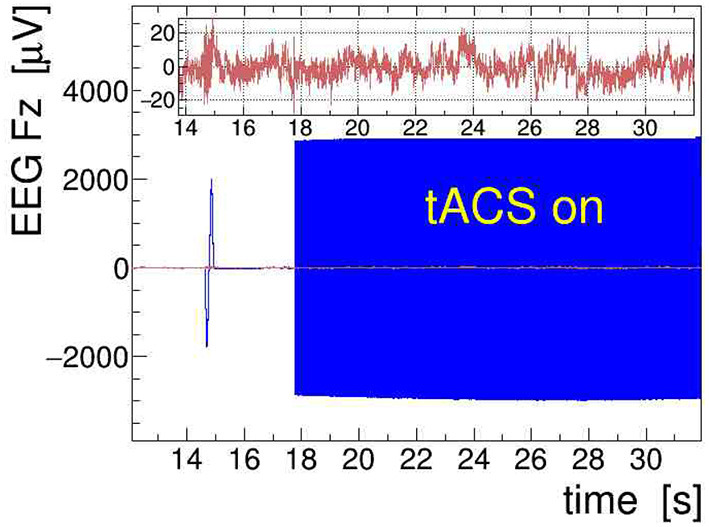
Removal of the main transcranial alternating-current stimulation (tACS) artifact from the electroencephalogram (EEG) signal. The signal recorded at site Fz is shown (in blue); at *t* = 18 s, a stimulation with 100 Hz is started producing a more than 100-fold increase of the signal amplitude. The clean signal obtained with the removal procedure introduced in section 3 is overlaid (in red) and also shown enlarged on the inset.

A sample of EEG data recorded during stimulation from channels Fz, ECG, and RESP is presented in Figure 9. The onset of stimulation with 100 Hz tACS is clearly visible in the EEG around time *t* = 18 s, with the electrophysiological signal becoming completely overpowered by the tACS-induced potential. Obviously, any quantitative analysis of EEG signals recorded during tACS application requires this nuisance effect to be removed with great care and precision. In particular, in cases where the EEG frequency band of interest is directly contaminated by artifactual power, application of digital filters, as done here on the ECG signal, is generally not a viable solution. We found, however, that the subtraction of the concurrently recorded stimulation current *I*_tacs_(*t*), applying Equation (11), provides a reliable and satisfactory means to remove the main tACS artifact. The result of this procedure is displayed in Figure 10 where the restored Fz signal is confronted with its original, contaminated instance. This direct comparison in the time domain offers already a good appreciation of the applied method; a more quantitative discussion in the frequency domain is given in the following subsection.

### 4.3. Performance of the Artifact Removal Procedures

**Figure 11:**
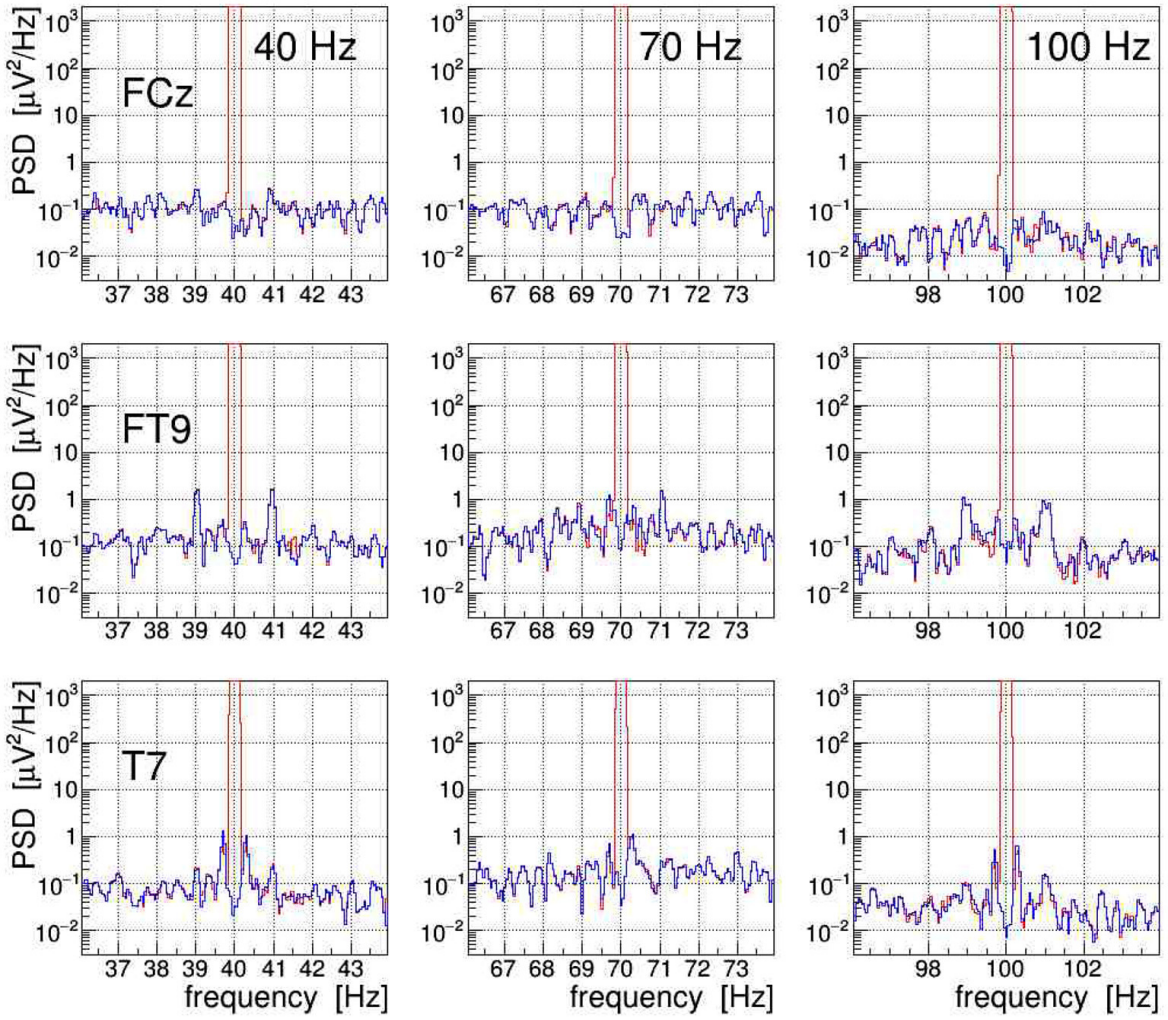
On-subject electroencephalogram (EEG) power spectra at the three electrode sites FCz, FT9, and T7 (from top to bottom) recorded during transcranial alternating current stimulation (tACS) application at one of the three frequencies 40, 70, or 100 Hz (from left to right). The power spectral density (PSD) obtained from 20-s episodes (0.05 Hz frequency bins) are shown for the uncorrected signal (in red) and for the signal with only the main tACS artifact removed (in blue). For at least two of these electrodes, one can clearly see amplitude modulation (AM)-induced side-band power, caused either by the heartbeat of the subject (FT9) or by his respiration (T7). For site FCz, the AM effects are also present but they are much weaker.

The efficacy of the applied tACS removal procedure can be better assessed in the frequency domain because there also weak contributions induced by the stimulation current can be easily recognized. The topographic map displayed in Figure 14A exemplifies the overwhelming electric signal produced by the stimulation current on all EEG electrode sites, particularly in close proximity to the tACS rubber pads.^7^ More specifically, Figure 11 shows a set of PSD distributions obtained by DFT (0.05 Hz bins, *α* = 4 Kaiser–Bessel window) of 20-s long episodes of the three signals FCz, FT9, and T7, which are recorded while stimulation current was applied at frequencies of either 40, 70, or 100 Hz. These three signals were chosen to exemplify the typical characteristics of the stimulation artifacts observed in the data and to also illustrate the performance of our removal algorithms. The PSD are shown for the uncorrected EEG (in red) as well as after removal of the main tACS artifact by applying Equation (11) to the signals (in blue). From this set of power spectra, one can see that for all stimulations applied, the cleaned FCz signal is basically artifact free while on electrodes FT9 and T7 prominent side-band peaks remain visible at about ±1 and ±0.3 Hz of the central frequency. The peaks corresponding to higher beat harmonics are expected to be very weak and, in our data, they are just barely visible above the intrinsic EEG baseline. The Δ*f* ≃ ±1 Hz frequency separation matches the heartbeat rate of the subject recorded at about 60–70 beats/min, and the Δ*f* ≈ ±0.3 Hz intervals agree with the recorded respiration rate of about 20 breaths/min. Our observations corroborate that, depending on the electrode site on the scalp, the EEG can be amplitude modulated by the heartbeat and the respiration at varying degrees of intensity. From the ratio of the side-band power to the main peak power, one can compute the modulation index *m* which characterizes the effect size. For heartbeat-induced modulation, we find values of *m* up to ≃ 1.0 × 10^−3^ and likewise, for respiration-induced modulation, up to ≃ 1.2 × 10^−3^. These values are compatible with the modulation effects reported by Noury et al. (2016), although the observed absolute side-band power differs between both experiments. Note, however, that differences in experimental conditions, in particular in the respective EEG montage used, in the placement of the tACS electrodes, in the intensity of the stimulation currents applied, but also (uncontrollable) inter-subject differences may explain these dissimilarities. Ultimately, all results concur in showing that the subtraction of the main tACS artifact is not sufficient to also remove the AM-induced side-band artifact.

**Figure 12:**
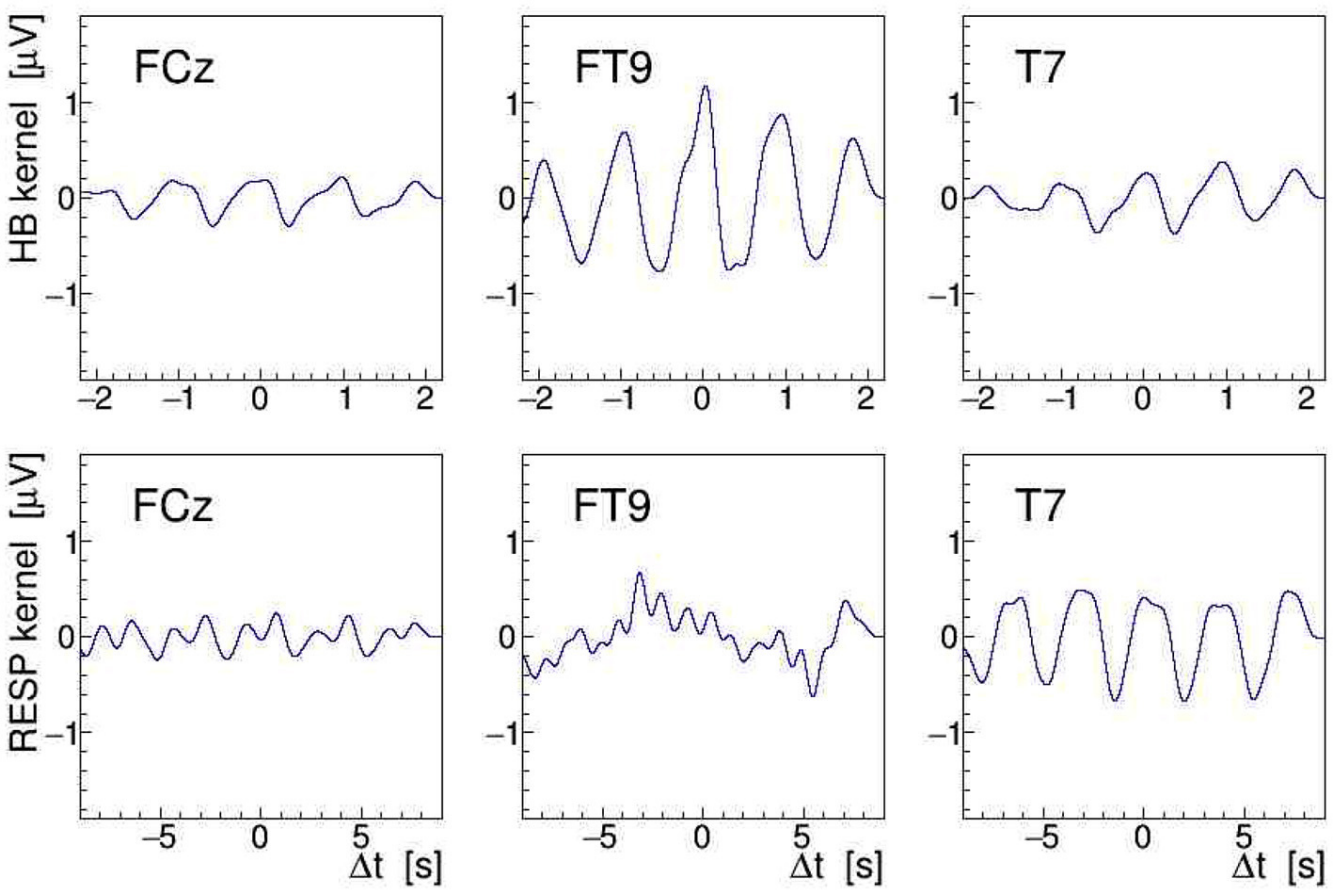
On-subject average amplitude modulation (AM) kernels $\bar{K}\left(\Delta t\right)$ obtained by demodulation of the electroencephalogram (EEG) signals recorded during 30 s of 100 Hz transcranial alternating current stimulation (tACS) application from electrodes FCz, FT9, and T7 (left to right). These kernels were obtained by averaging short segments of the signal envelope, time-locked either to individual heartbeats (upper row) or to individual breathing events (lower row). The $\bar{K}\left(\Delta t\right)$ are displayed as a function of relative time within the segments centered on the given electrocardiogram (ECG) or RESP events (see Figure 9).

Another, more direct way to quantify modulation effects is to demodulate the EEG signal at the given stimulation frequency (see section 2.2). To achieve this, we have computed the norm of its analytic signal by applying Equation (9) to the EEG. After detrending the result of this operation, needed to remove the constant term of the signal envelope, the AM kernel *K*(*t*) is obtained. As the modulation index is found to be of order ≤ 10^−3^ only, resulting in a very noisy single-event kernel, we have averaged *K*(*t*) over all heartbeats or respiratory events located within a given 30-s interval, and then low-pass filtered (32nd order Butterworth) the heartbeat-averaged and respiration-averaged kernels $\bar{K}\left(\Delta t\right)$ at 3.5 and 1 Hz, respectively; in this, Δ*t* stands for the relative time in the interval with respect to the heartbeat or breathing event. Here, it is important to realize that the event-averaging produces a kernel for either the heartbeat or the respiration, i.e., we end up with two separate kernels for each EEG signal. The $\bar{K}\left(\Delta t\right)$ extracted by demodulating and event-averaging the FCz, FT9, and T7 signals, recorded during a 100-Hz stimulation, are shown in Figure 12 as a function of Δ*t*. The amplitudes of the displayed kernels are a direct measure of the modulation effect size *m* and they confirm that the tACS signal on site FT9 is mostly modulated by the heartbeats while, on site T7, it is mostly affected by respiration.

**Figure 13:**
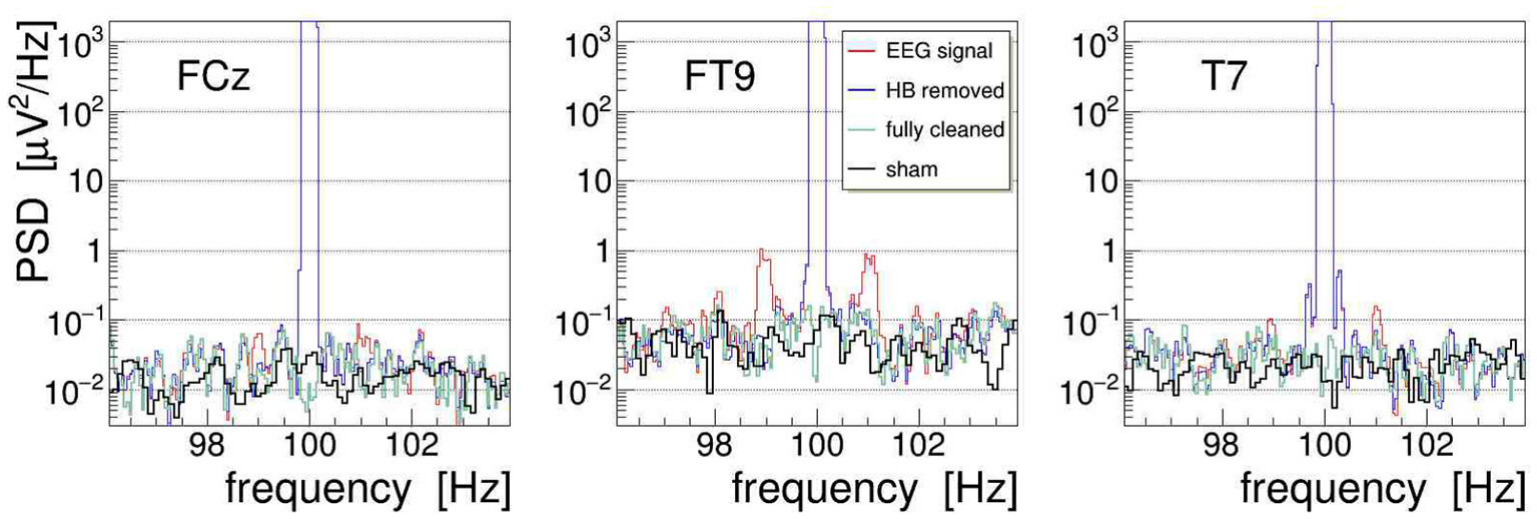
On-subject power spectral density (PSD) of the electroencephalogram (EEG) signals FCz, FT9, and T7 recorded during 100-Hz transcranial alternating-current stimulation (tACS) application (shown in red). The three artifact-removal steps discussed in the text have been applied, namely (1) removal of the heartbeat modulation (blue), (2) removal of the respiration modulation (not shown), and (3) removal of the main artifact at the stimulation frequency, yielding finally the fully cleaned PSD (green). For comparison, the fully cleaned PSD obtained after the end of stimulation, i.e., under sham condition, is also shown (black).

With the event-averaged kernels available, we can finally proceed to remove the amplitude-modulation artifacts from the measured EEG signals. Following the procedures outlined in section 3, we apply the corrections to each recorded EEG signal in three steps: first, we use the heartbeat-averaged AM kernel to remove, using Equation (10), the corresponding modulation from the EEG signal; second, we repeat this operation with the respiration-averaged AM kernel; and third, we suppress with Equation (11) the by far dominant artifact at the main stimulation frequency (by 85 dB for signal FCz, 83 dB for FT9, and 70 dB for T7). This cascaded cleaning procedure is exemplified in Figure 13 with the EEG signals recorded from electrodes FCz, FT9, and T7. Shown are the PSD distributions of the signals at different stages of the procedure: the PSD of the uncorrected signal is plotted in red, the PSD of the signal with heartbeat AM removed is shown in blue, and the final result, after removal of the respiration AM and the main artifact, is shown as green line. The figure also superimposes the respective PSD obtained during 20-s long off-stimulation episodes, i.e., under a “sham” condition (shown in black). Comparing with the artifact-corrected PSD, we find that the cleaning method is free of bias, comparable to our observations on the demonstrator.

**Figure 14:**
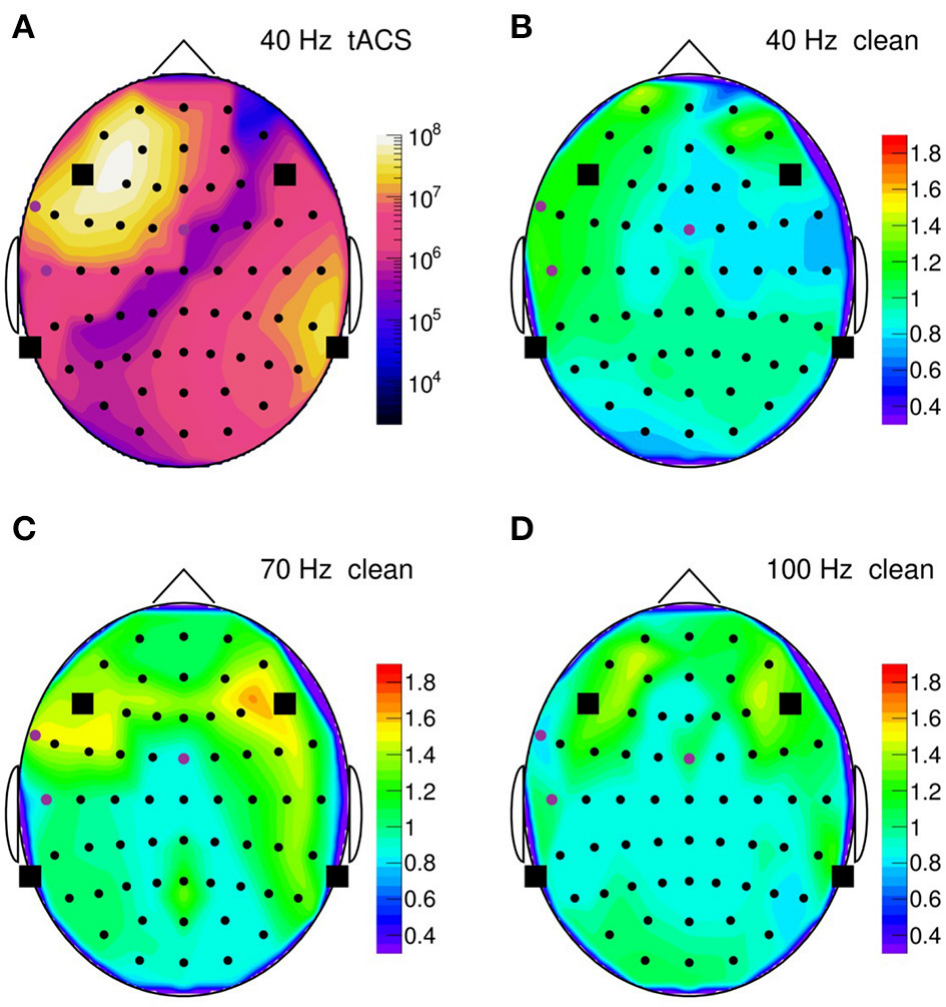
**(A)** Topographic map of the on-subject power spectral density (PSD) observed in the 35–45 Hz frequency band during 40-Hz stimulation, normalized channel-by-channel to the respective 50–70 Hz power; PSD is computed over a 30 s time span. Round symbols indicate the electroencephalogram (EEG) electrodes, squares represent the transcranial alternating current stimulation (tACS) electrodes. **(B)** Topographic map of the normalized PSD after artifact removal; **(C)** same for 70-Hz stimulation (normalized to the 80–100 Hz power); **(D)** same for 100-Hz stimulation (normalized to the 110–150 Hz power).

To summarize our results, Figure 14 presents topographic maps of the fully cleaned PSD obtained for a stimulation frequency of 40 Hz in **(B)**, 70 Hz in **(C)**, and 100 Hz in **(D)**. In generating these maps, the PSD has been integrated over a narrow frequency band, centered on the stimulation frequency, and normalized to a corresponding artifact-free frequency interval. This normalization intends to remove channel-by-channel gain and effect variations, resulting ideally in a clean PSD ratio of one. As Figure 14 shows, the ratio stays indeed close to unity (within 10–20%) on most of the scalp, demonstrating that the artifact removal works reliably over more than eight decimal orders of magnitude. However, in the fronto-temporal regions, a slight tendency to rise above one is visible and may point to a genuine increase of EEG activity induced by the stimulation. More studies are certainly required to clarify this point.

Note finally that, although we present all results in the frequency domain, the cleaning procedure itself is completely applied in the time domain. The fully cleaned EEG signal is hence also obtained in the time domain (see as well Figure 10), meaning that it can be subjected at will to any further analysis in either domain, time or frequency.

### 4.4. Limitations

In this paper, we have provided proof-of-principle results for a few stimulation frequencies only, 40, 70 and 100 Hz, all lying in the low and high gamma bands. This choice resulted from our observation that the side-band artifacts were very weak or even not observable for much lower frequencies, e.g., 10 Hz, in line with the overall rising trend of the intrinsic EEG power toward low frequencies. We see, however, no obvious reason why our cleaning procedures should not remain valid for stimulation frequencies situated in the lower bands. On the other hand, for very low frequencies (*f*_*s*_ ≪ 10 Hz), the small spacing between the overtones of the main artifact will eventually lead those harmonics to intermingle with the side-band frequencies. In other words, in cases where the artifacts would nonetheless remain visible, their spectrum would tend to develop a very complex structure, which then may become difficult to fully remove.

As already stated in section 4, we have kept the stimulation current at 0.6 mA_pp_. This was mainly motivated by our interest in applying combined tACS–EEG in sleep studies. In studies of sleep, the tolerance level for skin irritations, like tingling and burning sensations, as well as for induced visual effects (phosphenes) is usually quite low. However, these limitations may apply to a much lesser degree in many other investigations involving awake subjects. In that respect, the validation of our method would indeed have to be extended with higher stimulation currents. Still, all transformations involved in the cleaning procedure being linear, we do not expect the latter to fail when applied to larger currents, at least insofar as the EEG amplifier dynamic range can also accommodate the larger signals.

Another limitation of our experiment was that the subject kept largely immobile with eyes closed, again a setting typical for sleep studies. In investigations on awake subjects, in particular studies targeting cognitive and motor tasks (see, e.g., Santarnecchi et al., 2013; Guerra et al., 2018; Bologna et al., 2019), additional movement artifacts caused by eye saccades and blinking, head movements, body shifts, etc., can distort the recorded EEG. Unfortunately, because of their largely non-periodic nature, these artifacts are not directly amenable to a treatment with the cleaning algorithms discussed here. We believe, however, that our procedures can be combined with other cleaning methods, e.g., a modified eye blink detection and removal algorithm that synchronizes with the periodic stimulation current.

## 5. Summary

In this paper, we have discussed the nuisance effects induced in the EEG during application of transcranial alternating current stimulation confirming, in particular, the recent observation of amplitude-modulation effects by Noury et al. (2016). Assuming that *ad hoc* physiological mechanisms, involving the heartbeat or respiration, lead to rhythmic changes of the body impedance, these must in turn induce an amplitude modulation of the tACS artifact in the measured EEG signals. We have demonstrated how such modulation effects can be produced in phantom data recorded with a demonstrator setup. We have further described a multi-step artifact-removal procedure and validated its implementation on these recordings as well as on human subject data, while focusing on stimulation frequencies in the low and high gamma bands. In line with former observations, we were thus able to implement modulation effects artificially (phantom data) and confirm their existence in our human-subject recordings. However, the observed effect sizes turned out to be of lesser magnitude than those reported originally in Noury et al. (2016). We hypothesize that these differences are caused by dissimilarities of the used experimental protocols (e.g., distances between tACS electrode placement and EEG recording sites) as well as by inter-subject differences. It would be interesting to follow up on this line of thought with more systematic investigations, as this could lead to specific recommendations how to best minimize such artifacts in future studies. Our cleaning approach has, furthermore, the potential to lend itself to adaptive parametric filtering techniques, e.g., along the lines discussed by Kohli and Casson (2019). This will, however, require more dedicated investigations. The ability to monitor the actual impact of the stimulation on targeted neuronal circuits would be of great value, not only for basic science but also in the applied medical fields. In the clinical context, there is an emerging interest in tACS as a supportive treatment of various mental (Klimke et al., 2016; Elyamany et al., 2020; Kayarian et al., 2020) and movement (Krause et al., 2014; Castiglia et al., 2018; Felice et al., 2019; Guerra et al., 2020) disorders. While general guidelines for the application of electrical stimulations have been proposed (Antal et al., 2017; Lefaucheur et al., 2017), one should keep in mind that, in clinical settings, simple EEG protocols and robust analysis procedures, such as the one presented in this paper are to be preferred. To conclude, we believe that the results of the present study can help making progress into that direction.

## Data Availability Statement

The raw data supporting the conclusions of this article will be made available by the authors, without undue reservation.

## Ethics Statement

Ethical review and approval was not required for the study on human participants in accordance with the local legislation and institutional requirements. The patients/participants provided their written informed consent to participate in this study.

## Author Contributions

RH designed, implemented, and applied the analysis algorithms, and prepared the manuscript. JK-G supervised the EEG data collection. UV and AK contributed to the study design and critically reviewed the manuscript. All authors contributed to the article and approved the submitted version.

## Conflict of Interest

The authors declare that the research was conducted in the absence of any commercial or financial relationships that could be construed as a potential conflict of interest.

## Footnotes

^1^Again, for the sake of clarity, we have set all phase angles to zero. In the most general case, where φ_*k*_ ≠ 0, the expansion of Equation (3) will lead to additional terms in Equation (5) involving also the corresponding cosine functions. This restriction does, however, not curtail our line of argumentation.

^2^With a frequency binning of 0.05 Hz, this results in 0.4 Hz.

^3^We preferred generating pink noise over using pre-recorded EEG data in order to achieve a well-defined, reproducible drop of the noise power with frequency.

^4^A cleaning scheme following this line of thought has already been suggested in Noury et al. (2016).

^5^In the regression, the two parameters Δ*m* and Δ*t* were adjusted once per analyzed EEG signal and epoch, i.e., in total *N*_sig_ × *N*_epo_ times.

^6^One sample corresponds to 1 ms at the 1 kHz sampling rate used in the analysis.

^7^The corresponding power topographies for 70- and 100-Hz tACS look very similar to the presented 40-Hz map.
